# The impact of a terrorist attack: Survivors’ health, functioning and need for support following the 2019 Utrecht tram shooting 6 and 18 months post-attack

**DOI:** 10.3389/fpsyg.2022.981280

**Published:** 2022-10-25

**Authors:** Mark W. G. Bosmans, Carolien Plevier, Francoise Schutz, Lise E. Stene, C. Joris Yzermans, Michel L. A. Dückers

**Affiliations:** ^1^Netherlands Institute for Health Services Research, Utrecht, Netherlands; ^2^Municipal Health Services region of Utrecht, Utrecht, Netherlands; ^3^Norwegian Centre for Violence and Traumatic Stress Studies, Oslo, Norway; ^4^ARQ Centre of Expertise for the Impact of Disasters and Crises, Diemen, Netherlands; ^5^Faculty of Behavioral and Social Sciences, University of Groningen, Groningen, Netherlands

**Keywords:** terrorism, survivors, health impact, PTSD, health monitoring, long term, care utilization, qualitative

## Abstract

**Background:**

Extremely violent events such as terrorist attacks and mass shootings form a severe risk for the health and wellbeing of affected individuals. In this study based on a public health monitor, we focus on the health impact (including PTSD symptoms, physical problems and day-to-day functioning) of the Utrecht tram shooting, which took place in the morning of March 18th 2019. A lone gunman opened fire on passengers within a moving tram. Four people died, and six people were injured in this attack. The attack resulted in nationwide commotion and drew much media attention. Aim of this study was to increase insight into the health effects for the survivors (those directly impacted by a terrorist attack and the bereaved), and whether they received the needed care and support.

**Methods:**

Semi-structured interviews with accompanying questionnaires were conducted at six and 18 months post-attack. Overall, 21 survivors (victims/witnesses and loved ones of deceased victims) participated in the first series of interviews, 15 in the second series. Qualitative data were analyzed using reflexive thematic analysis, quantitative data was only described because of the low sample size.

**Results:**

At both six and 18 months after the attack many survivors had been able to resume daily life, and most rated their overall health as (very) good or excellent. At the same time, a substantial portion suffered from health problems such as posttraumatic stress symptoms and other complaints, and needed professional care. Furthermore, those in need did not always find their own way to appropriate care through the existing health system: half of the survivors still needed support in finding the right care 18 months later.

**Conclusion:**

Although the design and implementation of this public health monitor were accompanied by multiple challenges, it was possible to track a portion of the survivors and gain insight in the considerable health burden of the attack. Also, it is clear in this study that the health impact of terrorism affects survivors in the long run and requires attention from health authorities and professionals, as survivors were not able to find the right care by themselves.

## Main messages

- Many of the interviewed survivors reported a substantial long-term impact on health and functioning.
- At 18 months post-attack, half the interviewed survivors still needed help in finding help in finding the appropriate care.
- Challenges in the health monitoring approach were not new, and will occur again if registration of survivors and the exchange of their contact information are not improved.

## Introduction

Extremely violent events such as terrorist attacks and mass shootings form a severe risk for the health and wellbeing of survivors. Since the start of the 21st century, the number of terrorist attacks worldwide has been on the increase (The National Consortium for the Study of Terrorism and Responses to Terrorism, 2014, 2020). The same is true for mass shootings (Lowe and Galea, 2017). As a result, the need for accurate insight into the effects of these attacks on those involved has become ever more pressing. Before going deeper into the current study [the design and results of a public health monitoring program in the Netherlands, conducted in response to the Utrecht 2019 Tram Shooting (see Box 1), a mass shooting that was considered a terrorist attack by the Dutch court and EUROPOL^1^], we will go deeper into what is already known about the health impact and risk and protective factors after exposure to disasters in general and terrorist attacks and mass shootings in particular, and also what we can learn from conducting health monitoring programs.

From previous disaster studies we know that those exposed may develop problems such as anxiety, depression and posttraumatic stress (e.g., Norris et al., 2002; Bonanno et al., 2010; Hoven et al., 2012; Goldmann and Galea, 2014). From the same research we also know that a majority of those affected appear to have only a limited degree of problems, and that most recover on their own from any initial complaints. However, as Bonanno and Mancini (2012) demonstrated, a minority does develop long-lasting mental health problems. From previous disaster research, we also know that physical symptoms are more prevalent among disaster survivors than among the general population (Van den Berg et al., 2005; Yzermans et al., 2009). While the prevalence of these complaints generally decreases over time, the prevalence for some complaints can remain high for years post-disaster. In addition, among those exposed to disasters, the prevalence rates of diagnosed physical conditions – especially cardiovascular diseases – are also higher (Yzermans et al., 2009). Even though the distinction between mental and physical complaints is often made, with separate research lines, this distinction is, naturally, not always that evident. Often they are comorbid after disasters, with the lines between them not always clear: mental problems can express themselves through physical symptoms, and physical problems (especially when they are chronic or limit daily functioning) can lead to mental symptoms (Yzermans et al., 2009).

BOX 1 The Utrecht tram shooting took place in the morning of March 18th 2019 when a lone gunman opened fire on passengers within a moving tram. Four people died in the attack, and six were wounded. The shooter had a terrorist motivation for the attack (for which he was later also convicted; EUROPOL, 2020). Four people died, and six people were injured in this attack. The attacker was arrested after an extensive manhunt in the early evening of the same day. During the manhunt – which received national media coverage – public buildings and schools in Utrecht were closed, public transport was shut down, and citizens were advised to stay indoors.

When we look more specifically at the consequences of terrorist attacks, we know from previous reviews that their impact on the affected can be extensive. Among directly exposed (direct witnesses and those injured in the event), a large minority develops significant levels of posttraumatic stress symptoms (García-Vera et al., 2016; Stene et al., 2016; Rigutto et al., 2021), including PTSD. Other psychological consequences, such as (major) depression or anxiety disorders are also common among directly involved victims (Whalley and Brewin, 2007; Salguero et al., 2011; Dyb et al., 2014; Stene et al., 2016; Lowell et al., 2018; Rigutto et al., 2021). In addition, victims often suffer from physical complaints (e.g., Van den Berg et al., 2005; Yzermans et al., 2009; Stene et al., 2016; Glad et al., 2021). Among the indirectly exposed, the prevalence of problems is generally lower (Salguero et al., 2011; García-Vera et al., 2016; Rigutto et al., 2021). An indirect group still affected to a considerable degree consists of relatives and friends of the deceased (García-Vera et al., 2016). This is to be expected, as loved ones of deceased victims are exposed to a traumatic stressor (grief). The impact of the traumatic loss of a loved one can be great, and may result in mental health problems such as PTSD, depression and persistent complex bereavement disorder (Boelen et al., 2019). Victims and relatives might recover only slowly from their health problems (García-Vera et al., 2016). This makes this group very relevant when we want to understand the impact of such an attack. Other risk factors are the degree of exposure, demographics (e.g., female gender), pre-existing psychological problems, and low psychosocial resources (e.g., low social support; Salguero et al., 2011; Van Dijk et al., 2014; García-Vera et al., 2016). Since the risk profiles of survivors are not known, and because risk factors do not reliably predict outcomes for specific individuals, it is important to follow those affected by a terrorist attack over time in order to monitor their needs.

Other violent events whereby large groups can be affected are mass shootings, which are multiple, firearm, homicide incidents, involving 4 or more victims (Smart, 2018). While not all terrorist attacks are mass shootings, and not all mass shootings are terrorist attacks, they share the fact that one or several perpetrators aim to deliberately cause injury and death among groups of people, often random individuals. Central to both types of events is the malicious intent of the perpetrator(s). One may therefore assume that the impact of both types of event on the health of those involved can be similar. This is corroborated by reviews investigating the psychological impact of mass shootings that show that these shootings are associated with a range of adverse psychological outcomes, especially PTSD and depression and anxiety symptoms (Shultz et al., 2014; Wilson, 2014; Lowe and Galea, 2017). Risk factors are demographic factors (e.g., female gender), pre-incident characteristics (e.g., pre-existing psychological symptoms), event exposure (greater proximity to the attack and acquaintance with the deceased) and fewer psychosocial resources (e.g., lower social support; Shultz et al., 2014; Wilson, 2014; Lowe and Galea, 2017).

From previous research into utilization of mental healthcare after disasters, we know that despite the burden on mental health, some disaster victims are reluctant to utilize mental healthcare services; even among those with severe mental problems there are large groups who go untreated (e.g., Whalley and Brewin, 2007; Rodriguez and Kohn, 2008). In other words, there might be a group which does not get the support and/or care it needs. There are examples of a much higher (mental) health care utilization (e.g., after the Utøya attack in Norway, Stene and Dyb, 2015), yet these are associated with proactive outreach programs and a well-organized (and accessible) healthcare system. Barriers to getting appropriate care after a terrorist attack are factors such as fear of stigma, financial constraints, a lack of mental health literacy but also a perceived lack of expertise of those who offer psychosocial care (e.g., Rodriguez and Kohn, 2008; Van Overmeire et al., 2021; Stancombe et al., 2022). These findings indicate that service delivery after terrorist attacks may be inadequate, or at least that the barriers that are commonly present hinder victims in accessing available care.

As has been noted before (e.g., North and Pfefferbaum, 2002), conducting methodologically sound health research after any disaster, but especially after a terrorist attack, is very difficult. While there are of course issues of the ideal timing of measurements and the type of measurements conducted, as is the case after most disasters, some of the major challenges center around the registration and sampling of those affected (e.g., Dyregrov et al., 2019; Jacobs et al., 2019; Stene et al., 2022). A terrorist attack usually takes place in a public setting with a high number of people present, many of whom will try to flee the scene of the attack. Registration systems are usually not in place, and priorities of first response lie elsewhere. Moreover, often large numbers of rescue workers and law enforcement and/or armed services personnel are called in to deal with the direct aftermath, exposing them to horrific scenes. Furthermore, terrorist attacks are usually accompanied by media coverage that may lead to fear for the safety of loved ones, exposure to shocking images, and fuel distress (Thompson et al., 2019). This makes it very hard to determine who was affected by the event. Not only is it complicated to get an accurate picture of the actual size of groups (either directly and indirectly) impacted by the attack, the registration of victims in order to include them in research at a later time point is fraught with challenges. One of the core questions for researchers conducting measurements after a terrorist attack is how to get a representative sample of those affected, and what the exact denominator (total number of affected) is (Bosmans et al., 2022).

The aim of this study was to increase insight into the health effects for the survivors (those directly impacted by a terrorist attack and the bereaved), and whether they received the care and support needed in the medium-and long-term. Despite the difficulty of registering direct victims of such an event, we were able to include a sample of those most directly impacted by the event either by personal proximity or by acquaintance with deceased victims; including both injured victims, direct witnesses and relatives of fatal victims. This study adds to the existing literature by (a) describing the design and implementation challenges of a public health monitoring program in the Netherlands, conducted in the wake of the 2019 tram shooting, and (b) by giving a detailed – qualitative – insight into the impact of this terrorist attack on the lives of those most directly affected in the medium-and long-term.

## Materials and methods

### Interviews

Data was gathered using semi-structured interviews with an accompanying quantitative survey conducted by experienced specialized trauma psychologists at six and 18 months post-event. Topic lists were composed by trauma and disaster experts at the Netherlands Institute for Health Services Research (Nivel) and ARQ National Psychotrauma Centre. The main topics during these interviews were: the wellbeing and health of the affected; what care and support was used; whether further support or support of a different kind was wanted; what factors impacted recovery and the evaluation of meetings organized by the municipality and other manifestations of support. Duration of the interviews ranged between 37 and 127 min (mean 80 min). A copy of the questionnaires (in Dutch) can be obtained from the second author.

Interviews during the first measurement were conducted at the participants’ home (17), at the municipal health services office (three) or at the participants’ place of work (one). The interviews had some aspects of an intervention: the professionals explained what normal reactions to experiencing such an abnormal event are. In 11 of the 21 interviews, advice was given on fitting care in the first round of interviews. In the second round this was the case for seven out of 14 interviews. In addition, some interviewees were brought into contact with other survivors, the police or with municipal services upon their request. The professionals also helped a number of survivors with finding the most appropriate care by drafting a letter for general practitioners (GPs) or by giving contact information for specialized care. During the second round of interviews, the COVID-19 pandemic had started. Because of this, precautions were taken during the face-to-face interviews (such as keeping distance), and 5 out of 15 interviews were conducted using Microsoft Teams, an online meeting application. Also, questions were added to the second interview regarding the influence of the pandemic. These focused on infection with the virus, impact on physical and mental health, on lifestyle and on one’s financial situation.

### Participants

**Figure 1:**
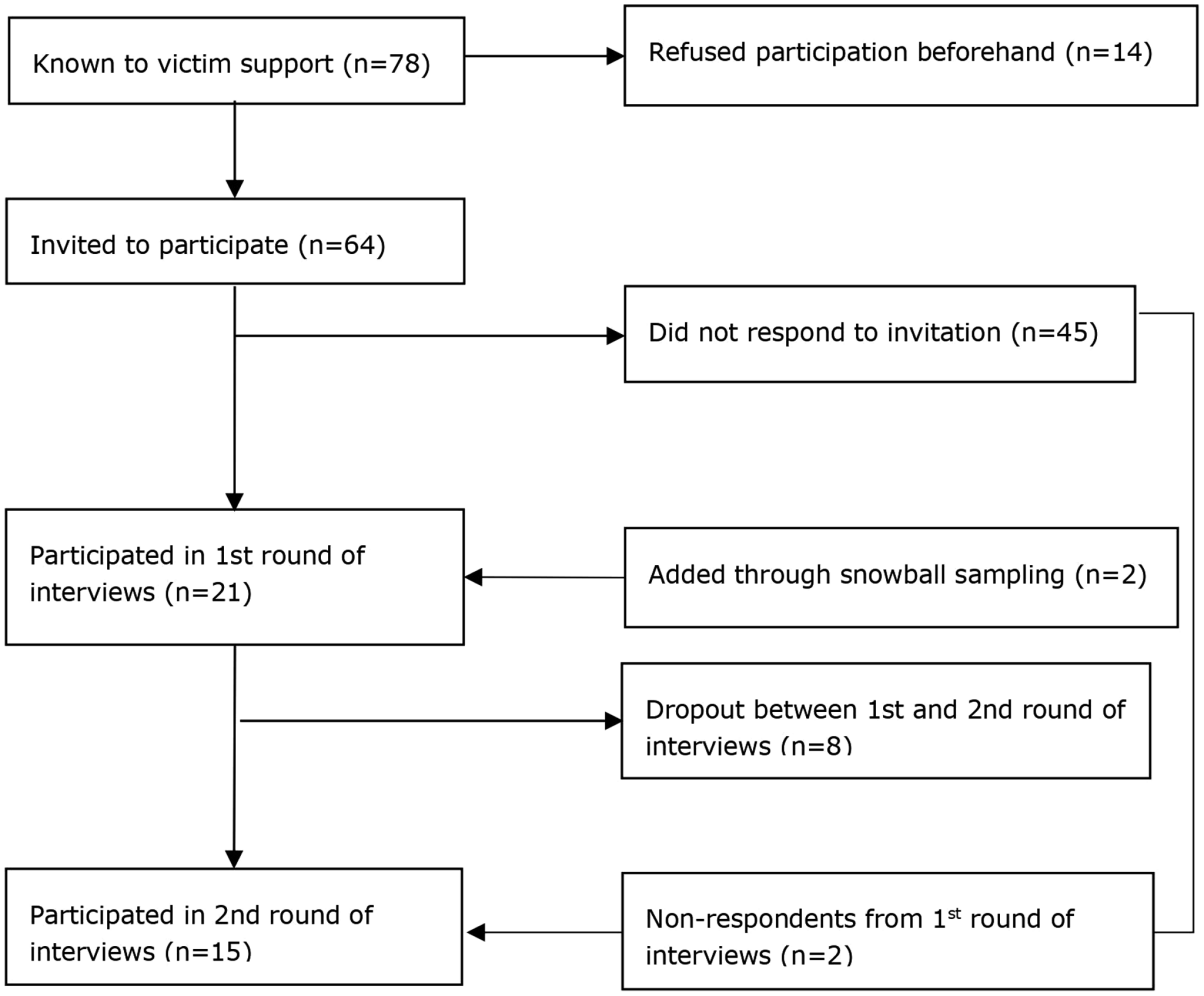
Flowchart of study enrollment.

Participants in this study were those who were involved in the shooting either as a survivor, a close relation of one of the deceased, or as a direct witness. There were 78 affected individuals known to victim support services. Of these, 14 had indicated they were not willing to participate beforehand. 64 affected individuals were invited to participate in the study of which 19 participated in the first interview. Two additional respondents were added through snowball sampling (these witnesses were not known to Victim Support, but were known to participating respondents), resulting in a sample size of 21 for the first measurement at 6 months post-event. Of those, 13 participated in the second round, with an additional two respondents who had refused participation in the first round, resulting in a sample size of 15 for the second measurement at 18 months post-event (see Figure 1).

### Measures

The following standardized instruments were used in the quantitative survey:

#### Impact of events scale-revised

In order to measure posttraumatic stress symptomatology, the Dutch version of (Brom and Kleber, 1985) the Impact of Event Scale-Revised (IES-R, Weiss and Marmar, 1997) was used. This 22-item instrument measures three symptom-domains of PTSD: reexperiencing, avoidance and hyperarousal. The IES-r has been extensively validated in previous research, where it was demonstrated to have high construct validity and reliability (Briere, 1997; Weiss and Marmar, 1997; Creamer et al., 2003; Beck et al., 2008). Cronbach’s alpha’s for the IES-r were excellent at both waves (*α* > 0.96). We used a cut-off of 36 to determine whether an individual had probable PTSD.

#### Symptoms and perceptions questionnaire

Physical symptoms were measured using the Symptoms and Perceptions (SaP) questionnaire (Yzermans et al., 2016). This instrument measures prevalence, perceived seriousness and duration of physical and psychological symptoms experienced in the last month. For this study a short version was used with 22 items assumed to be relevant in the aftermath of the shooting.

### Analysis

The qualitative portion of the interviews were transcribed verbatim and analyzed using MAXQDA (VERBI Software, 2021), and were coded and analyzed using reflexive thematic analysis. The quantitative data from the standardized instruments is only described. Because of the low sample size, statistical testing of differences was not performed.

## Results

In the first interview round which took place 6 months after the attack, 21 people participated (15 present at the scene of the attack (including wounded and eye-witnesses), and six family members of fatal victims), in the second round of interviews at 18 months after the attack this was 15 people (11 present at the scene of the attack and four family members). The majority of respondents were men (12 out of 21 at 6 months, and nine out of 15 at 18 months post-attack). Almost all respondents were between 18 and 65 years of age (see Table 1).

**Table 1:** Descriptives.

	1st measurement	2nd measurement
*Sex*		
Men	12	9
Women	9	6
*Age*		
<18 years	1	–
18–30 years	6	4
31–50 years	8	5
51–65 years	3	5
>65 years	3	1
*Exposure*		
Present at scene of attack	15	11
Family member of victim	6	4

### Outcomes first round of interviews – 6 months post-event (*N* = 21)

#### Symptoms of posttraumatic stress

A number of the study participants indicated that they had been diagnosed with PTSD by a professional. When we looked at the outcomes from the posttraumatic stress measure in the interviews, this was corroborated: 16 out of 21 study participants had a score above the cutoff for the IES-R. In other words, it is highly likely that a substantial portion of the investigated group suffered from substantial posttraumatic stress 6 months post-event:

*I am hyperalert now: I pay much more attention when I am in a public setting. I did not have that before.*

#### Other psychological problems

In the interviews, other psychological problems and stressors were also reported by respondents. Almost all respondents experienced sleeping problems: 10 of them had severe sleeping problems at the time of the interview, and another six had moderate sleeping problems. For three of them this concerned pre-existing problems. Problems with concentrating were also mentioned by six respondents as a result of the attack. Respondents also reported multiple emotional consequences of the attack, such as numbness:

*I am not doing so well, have completely no emotions anymore. Totally flat, not able to really feel.*

Eleven respondents indicated to be angry very often since the attack. Usually anger towards the perpetrator, but also anger towards the justice system or towards fate:

*I can become angry so quickly, for the slightest thing. When I drop something, I can become really angry, completely demolish the house so to speak. I never used to have that.*

Fear was also mentioned by 11 respondents, all of them present at the scene during the attack. For them, fear continued to play an important part in the period since the attack. Five respondents indicated to be afraid of experiencing a similar event again. Almost half (10) of the respondents frequently experienced grief. Five of them had lost a loved one in the attack. Other reasons for grief were: guilt and not being understood by friends and family. Guilt was also commonly experienced by respondents. Guilt for instance for not being able to prevent other from being shot, or for having to run for their lives and being unable to continue to offer aid to others:

*Rationally, you know that you could not have done anything. That you have not been negligent, that you did not fail, that you could not have done anything. But inside it feels completely different.*

Positive emotions were also shared during the interviews, such as pride and gratitude. The feeling to have made a difference during the attack gave respondents a positive feeling. Some were able to help others to safety, called the police, or warned approaching motorists and cyclists away from the scene of the attack. Respondents were grateful for the help they received from others present at the attack, and also for the support received from their friends and family since the attack. Two respondents indicated that a positive change they experienced in addition to the negative consequences was the feeling to be living in borrowed time, which led to setting new life priorities.

#### Physical and psychological symptoms

Physical problems related to the attack (such as musculoskeletal problems, problems with the digestive system or fatigue) were mentioned by eight respondents, either as a result of injuries or as a result of stress. Furthermore, nine out of 21 respondents indicated that their health problems had worsened since the shooting (see Table 2). Additionally, respondents were asked about specific physical and psychological symptoms experienced in the last year, as well as whether these were already present before the attack. Half the respondents indicated that they experienced new health problems as a result of the shooting. The most common of these new symptoms were: psychological problems, fatigue, cardiovascular symptoms and problems with the digestive tract.

**Table 2:** Physical and psychological symptoms.

Symptoms	Measurement 1 (*N* = 21)	Measurement 2 (*N* = 15)
Digestive tract	52% 11	33% 5
Cardiovascular system	43% 9	40% 6
Fatigue	62% 13	80% 12
Nervous system	62% 13	73% 11
Musculoskeletal system	62% 13	73% 11
Respiratory tract	38% 8	33% 5
Psychological	76% 16	100% 15
High level of posttraumatic stress symptoms	76% 16	53% 8

#### Overall health

Respondents were asked how they experienced their overall health. Twelve out of 21 respondents indicated that their health was (very) good. Another three respondents indicated to have poor overall health.

#### Impact on wellbeing and functioning

When asked to rate their wellbeing, the average grade was just below five (out of 10). 19 out of 21 respondents did indicate that they had resumed their daily activities such as work or school. Five respondents indicated that they made arrangements at work or school regarding adapted tasks and/or hours. Four respondents indicated that the shooting had a negative impact on their finances, either by losing their job or by not being able to work (for the self-employed). Those who were unemployed before the shooting still had no job 6 months post-event. Four respondents also indicated that they experienced problems in their relationship with their spouse/partner as a result of the shooting. Finally, eight of the respondents indicated that the shooting impacted family members (who were not present at the attack) as well.

*We will probably never again be the same as we were on March 17^th^.*

#### Factors that impacted processing of the attack

A very important factor reported by the respondents was receiving recognition as a victim. Some of the respondents felt that professionals did not fully acknowledge the impact of their experiences. Two respondents were also deemed not to be victims in the legal sense, which led to frustration and grief.

*A case manager entered. She said she was only case manager for the severe cases like I…..well, I’m here, but really I’m only for the severe cases.*

Another factor that had a great impact was the cumulation of problems among the majority of the respondents. 15 out of 21 respondents indicated that they had experienced traumatic events before the attack. The media also impacted recovery for some. There was a lot of media-attention for the event, and its aftermath, with high media presence at events such as the silent march to remember the victims. The presence of the media impeded attendance of meetings with fellow victims or of remembrance gatherings for some respondents.

*I’ve resumed traveling by tram. In the beginning, it was really difficult for me to go there, because everything was still fresh. I was afraid that if I started to cry, a camera or microphone would immediately be pointed my way.*

A final factor mentioned in the interviews was the fact that the perpetrator was of Turkish origin (born in the Netherlands). Some of the respondents feared that society might look at them askance because of their own migrant background. Several respondents also mentioned that they were brought to safety by other victims with a migrant background, and regretted the fact that there was no media attention for this.

#### Care and support

Out of 21 respondents, 19 were in contact with Victim Support after the tram shooting at least once. Their experiences were diverse. Some survivors were very positive and experienced much support from their contact with Victim Support. Others indicated that their contact with Victim Support was very brief and sporadic, and that there was no follow up.

Out of 21 respondents, 17 indicated that they visited their GP after the attack, and for 16 of them this visit was related to the attack. Additionally, 14 of the respondents indicated that they had been in contract with a psychotherapist, psychologist or a psychiatrist as a result of the attack. More than half (11) the respondents received help with processing the event. The other 10 did not receive help at the time of the first interview. Of these, four indicated that they did need help. Seven of the 21 (one of which did receive help) indicated that they did not need help or support with processing the event. Of those who did receive help at the time of the first interview, four indicated that this was either not sufficient or not appropriate. Of those with a high level of posttraumatic stress symptoms, almost half did not receive care. Five respondents also indicated that they found it difficult to receive the right kind of care. Reasons for this were for example unfamiliarity with the (mental) healthcare system, not knowing what kind of care they need, or (real or perceived) financial constraints. In 11 out of 21 cases, the trauma psychologists conducting the interviews gave advice with regard to finding the appropriate care, indicating that not everyone had yet been able to find fitting care at 6 months post-event:

*Now, since a month and a half, I receive psychological help. I notice that my mental processing is only now beginning.*

*I have been referred to a psychologist, there was a waiting list of a year. I then sought another, am now on a waiting list.*

### Outcomes second round of interviews – 18 months post-event (*N* = 15)

#### Symptoms of posttraumatic stress

Based on the IES-R, half the respondents (eight out of 15) have a score above the cutoff, indicating possible PTSD. The scores of 11 of the (13) affected who participated in both interviews (at six and 18 months post-attack) remained the same between the first and second interview. Of these 11, five scored below the cutoff, and six above. For the other two affected who participated in both interviews, the scores on the IES-R improved.

#### Other psychological problems

Five respondents indicated that they suffered from sleeping problems as a result of the attack. They also suffered from nightmares in which they re-experience images from the attack. Two respondents indicated to still be troubled by symptoms of re-experiencing, and one indicated to be hypervigilant in busses or trams. Three respondents indicated to be worried by the fact that they have become more fearful since the attack. Finally, three respondents suffered from concentration problems:

*When I’m with my mother, my mind strays very quickly. I am there, but also not there. My thoughts are simply gone. I don’t know what goes through my mind then.*

Other commonly experienced problems are stress-related complaints: seven respondents indicated that they regularly feel stressed. This is partly due to the consequences of the attack, but also due to their personal situation:

*In the train I now sit in a corner at the rear of the train upstairs, or directly by the door or the hallway, so I can monitor everything. If anything happens I can leave immediately.*

*If things come up unexpectedly or if things do not succeed, I get really stressed and very irritated.*

18 months after the attack, several respondents indicated that they would like to leave the attack behind them, and move forward with their lives. This come up in eight interviews:

*It happened to me, and we will move on. I do notice that I’ve started to appreciate life a little more.*

#### Physical and psychological symptoms

Respondents were asked about specific physical and psychological symptoms experienced in the last 6 months, as well whether these were already present before the attack. The high number of respondents with psychological symptoms (all respondents) and fatigue (12) are the most striking (see Table 2). Symptoms related to headaches and dizziness and problems of the joints and muscles were also common. According to the respondents, most of these symptoms were due to the attack.

*My health is much better than last year of course. I still train with the physical therapist once a week.*

*My health is mediocre, because I have aches and pains everywhere, which I did not have before the attack.*

#### Overall health

When asked to rate their overall health, nine respondents indicated that their health was (very) good or excellent. Another five indicated that their health was mediocre, and one respondent that it was poor. When asked to compare their current health to that before the attack, three of the respondents indicated that it had worsened. Two respondents indicated to have health problems that they had not experienced before the attack. Of the respondents who participated in both rounds of interviews, three indicated that their health had improved, three that their health had deteriorated, and six that their health had remained the same.

#### Impact on wellbeing and functioning

During the second round of interviews, respondents rated their life with an average score of 6.3 (out of 10). Of the 15 respondents, six gave their lives an inadequate score. The respondents who took part in both interview rounds (13) gave an average score of six at the first interview, and a 6.1 at the second one. Crucially, the average grade of those who only participated in the first interview round was 3, indicating that those who did not take part in the second round of interviews had poorer life quality than those who did. The respondents compared their current overall situation (health and wellbeing, lifestyle, work, social contacts, financial situation, etc.) with that of 3 year ago, i.e., 6 months post-attack. Of the 15 respondents, eight indicated that their current situation had improved. Four others indicated that their situation was the same, and three that their overall situation had deteriorated. Eleven respondents worked or received education or schooling at the time of the interview. For three respondents their situation had changed since the first interview: one changed employers, one could not work because of current intensive psychological treatment, and one was working with a guidance counselor on career choices.

#### Behavioral changes

In the interviews, some behavioral changes were also mentioned: three respondents started smoking more after the attack, and five respondents started drinking more. On the other hand, one of the respondents stopped using cannabis after the attack.

*When I get home, I usually drink four beers, because, yes, I cannot relax otherwise. Because you feel irritable all day long. Like you are continually in highest alert.*

The way in which survivors approached the site of the attack differed widely. Several respondents indicated that they still regularly passed the site of the attack, and sometimes even visited the memorial, while others avoided the site completely:

*Until today I cannot bring myself to visit the place where it happened. I avoid that. When I need to go to the unemployment agency, I definitely will not pass the 24 October Square.* ^2^

#### Care and support

Eleven respondents did receive care or support to help process their experiences of the attack or deal with its consequences (see Table 3). Respondents most often received care from a psychologist or psychotherapist or from Victim Support. Out of these 11, four still received care at the time of the second interview. Six respondents received EMDR-therapy. This helped with processing the experiences of the attack for some of them. However, two respondents indicated that they found the therapy very burdensome, causing them to end it prematurely. Twelve respondents felt that they had received the appropriate care and support to help them with processing the attack. Eight respondents indicated that they still needed care or support. Of these, half (four) still received help, and the other half did not.

**Table 3:** Use of professional support in the 6 months prior to 2nd round of interviews (*N* = 11).

Care provider	Healthcare use in last 6 months	Healthcare use related to attack
General Practitioner	6	3
Psychologist/psychotherapist/psychiatrist	9	9
Victim Support	6	6
Social worker	2	2
Occupational physician	4	4
Specialist in Hospital	7	2
Practice nurse	2	2
Other	1	1

Finding the right care and support had been a challenge for several respondents. An important factor in this was the feeling of being understood. The treatment by care providers was crucial for this. Other reasons for the struggle with finding the appropriate care or support was related to the waiting lists in (mental) health care. In other cases, respondents first wanted to deal with their problems on their own, only to find out later that they could not. This was mentioned in five interviews. In all of these cases, the respondents indicated that they realized that they needed to find help.

*The general practitioner called me after the attack. That didn’t jive so well. If somebody says to me: “you feel this and this” I think: wait a minute. I am talking to you on the phone. I haven’t said anything, and you already know how I feel.*

Victim Support helped the affected with preparing for the court proceedings and with petitioning for compensation. This support was highly valued by the respondents. Some were less satisfied about the support by Victim Support, the municipality and other service providers, who felt that more intensive guidance would have helped them find fitting care sooner. This group, who often had a cumulation of vulnerabilities and a lack of social support might benefit from more intensive guidance and support. Targeting them would require including these vulnerabilities in the initial assessment of the affected.

*From the municipality and relevant organization it would have been nice if support was offered more pro-actively. We had to organize much ourselves.*

*It should have been written in the government protocol that survivors should be visited after three or four months.*

*I feel that I won’t manage on my own like this. So yes, how do I cope with my current situation in which I’m walking on eggshells and looking forward to some light at the end of the tunnel?*

Just like in the first round of interviews, the trauma psychologists conducting the interviews helped half the interviewees in finding the right care. This clearly signifies that even 18 months post-attack, a substantial portion of the affected still needed support in finding the right care and/or support.

#### COVID-19 pandemic

None of the respondents indicated that they believed to have been infected with the coronavirus. Some respondents did indicate that the pandemic has led to changes in their lives and their health. More stress, feelings of anxiety, depression or loneliness were most often reported. The majority of respondents however, indicated no change as a result of the pandemic.

## Discussion

### Impact

This paper extends our understanding of the long-term impact of terrorist attacks and mass shootings on those most affected, including those who were wounded and loved ones of deceased victims. The picture of this impact is two-sided at both six and 18 months post-attack. At 6 months post-attack most of the study participants had resumed their daily activities such as work or schooling. This is a clear demonstration of the resilience of the survivors. Nevertheless, the attack had a considerable impact on their lives. This is made clear by the high proportion of survivors with high levels of posttraumatic stress symptoms and physical health problems. One year later, at 18 months post-attack, the picture was comparable: many had been able to resume their daily life, and most rated their overall health as good, very good or excellent. They had been able to process the attack and were ready to move on. At the same time, a substantial group continued to suffer from a high level of posttraumatic stress symptoms. A large portion also suffered from long-term health problems, most of which originated after the attack. Furthermore, finding the appropriate care remained difficult: half of the survivors still needed support for this from the trauma psychologists conducting the interviews. Interestingly, the ones who were most directly affected (injured or came face to face with the perpetrator for instance), are not necessarily the ones who were most severely impacted. The interviews showed that degree of social support, history of previous traumatic events, and coping mechanisms used contributed strongly to recovery.

The fact that most respondents were able to resume their daily functioning, and that most rated their overall health as good or excellent despite the fact that most had physical or mental problems, is a clear illustration of the fact that people are not the sum of their problems. Many people are able to continue functioning despite physical or mental problems. This does not mean that they do not need or seek help, but it is a clear indication that one should be careful not to overly medicalize those who are exposed to traumatic events. The focus should not be solely on symptomatology such as posttraumatic stress without looking at the person and their surroundings. Having symptoms does not mean one is automatically a patient. In the end, the choice of seeking help for the symptoms someone has is ultimately their own.

### Registration of survivors

In this study we were able to gain insight into the impact on people with different levels of exposure to a terrorist attack. Yet, the small size of our sample precludes conclusions about the impact on affected of terrorist attacks in general. An important factor that led to the sample size, and that forms an impediment in most post-disaster studies, is that we did not have a full registration of all those affected by the attack. This precluded getting a full picture of potential target groups, for both interventions and monitoring. This was caused by the lack of an existing joint registration approach by the parties involved in the aftermath, and the fact that legal and privacy issues hindered the exchange of contact (and other) information. These two factors, in combination with the fact that not one single organization is made responsible for the coordinated service delivery for survivors and bereaved families in the aftermath, hinder the public health response in general and health monitoring in particular. These are not new problems (Jacobs et al., 2019) nor are they typically Dutch (e.g., Dyregrov et al., 2019; Stene et al., 2022). These problems are bothersome, and demand attention as it is a common finding across countries that the registration of victims, monitoring their health and evaluating the (psychosocial) care that is offered are crucial elements in providing the most appropriate care (Te Brake and Dückers, 2013; Bosmans et al., 2022; Dückers et al., 2022). It makes sense to resolve these issues not only nationally but also internationally (Stene et al., 2022), under the realization that different professions and institutions will be involved in different countries.

### Care and support

Despite the fact that the majority of respondents had been in contact with a health professional to deal with the consequences of the attack, there was still a majority that felt that they needed (more/other) care 18 months after the attack. Of this majority, half did receive help at the time of the interview, and half did not. Some reported that this was due to the fact that the care they did receive was not fitting care or that care was offered too soon after the attack. For others the delay was due to the fact that they did not feel they needed help initially, but realized this in a latter phase. However, long waiting lists also played a part. All in all however, these results suggest that the care and support offered were either not sufficient or did not reach the intended target group sufficiently or at the right time. Perhaps appropriate care was not available for the interviewed survivors because they experienced different phases of problems and needs over time, including new life events.

Of course we should mention here that the low sample size of this study means that we cannot extrapolate these findings concerning the number of people unable to find the appropriate care to the general population or even to all affected of the Utrecht tram shooting. These findings do demonstrate however, that it is important to keep monitoring those impacted most directly by terrorist attacks (direct victims/witnesses and family members of victims) for any needs or problems that may develop later. An appropriate measure for this would be conducting rapid needs assessments (Korteweg et al., 2010; Bosmans et al., 2022). Such assessments need not be extensive or time consuming, but can give valuable insight into the development of the development over time of the needs and problems of survivors and, crucially, into the effectiveness of disaster response services.

In theory, there are few barriers in seeking care in the Netherlands. On paper, professional care is accessible for everyone in the Netherlands. Specialized (psychological) care can be obtained through one’s general practitioner (GP; in the Netherlands, there is a regular GP scheme with full gatekeeping, which means that all specialized care is accessible only through a referral by a GP), and is covered by health insurance. In practice however, there are several barriers against seeking help. Some of these are common to disasters globally such as: not realizing one has a (severe problem) or believing one can solve the problem on their own, lack of knowledge about where to obtain help, concern about stigma, concern about costs, belief that treatment is ineffective etc. (Rodriguez and Kohn, 2008). Specific barriers to care in the Netherlands are that there are long waiting lists for (specialized) mental healthcare (Vektis, 2022), and that individuals who suffer from complex or multiple mental health problems can have trouble finding appropriate care (Te Velde et al., 2018; Onderzoeksraad voor Veiligheid, 2019; Inspectie Gezondheidszorg en Jeugd, 2021). Finally, in the Netherlands access to specialized mental healthcare depends on the decision by the GP that one is indeed in need of such specialized care. This is more than a mere formality. Because of a policy change that took place in the Netherlands in 2014 aimed at cutting costs of specialized mental healthcare, the role of general practice in mental healthcare has greatly increased. As a result, the GP and general practice nurses specializing in psychological problems see many more patients for psychological problems than before. In fact, the majority (75%) of patients with mental health complaints in the Netherlands are treated within the general practice (Magneé et al., 2018).

As has been demonstrated in previous research, the organization of care and support after a terrorist attack varies widely between countries (e.g., Stene et al., 2022), not only with regard to the parties involved, but also in the degree to which an outreach approach is used whereby survivors are actively approached to refer to care. International guidelines recommend the monitoring of needs and problems of the survivors of disasters (Inter-Agency Standing Committee (IASC), 2007; Bisson et al., 2010; Te Brake and Dückers, 2013; Juen, 2016; International Committee of the Red Cross (IFRC), 2018; Dückers et al., 2022). However, such monitoring is only meaningful if it is followed-up by general or specialized health care professionals when needed.

### Limitations and strengths

The sample size of this study was quite small. Furthermore, the response rate among those known to Victim Support or the public prosecution service was not very high. As a result, we should be careful in extrapolating our findings to all affected. Results might not be fully representative of the entire affected group. What we can do with this study is describe how those who did participate fared in the 18 months after the attack. The fact that we used semi-structured interviews conducted by specialized trauma psychologists, means that the information gathered went beyond standardized clinical instruments, and allowed gaining a deeper understanding of the way the lives of those involved are affected. In these interviews, the impact of the event could be measured in more detail, which offered much richer information about the impact of the attack on the lives of survivors. Furthermore, our small sample was comprised of people with a high degree of exposure to the attack, including both wounded victims and next-of-kin of deceased victims. Because respondents came from different groups, such as direct victims, relatives of deceased victims and eyewitnesses, coupled with the use of both quantitative and qualitative measures, we were able to get a picture of the diversity of the impact of an event such as this. We were also able to identify the factors that play an important role in the recovery process. Finally, this study gives a clear picture of the possibilities for improvement when it comes to the care and support offered to victims of terrorist attacks.

Another limitation is that we did not conduct diagnostic interviews to assess PTSD. The gold standard is a diagnostic interview conducted by a clinician using the Clinician-Administered PTSD Scale (CAPS; e.g., Hunt et al., 2018). Instead we used a screening instrument only intended for population screening. Nevertheless, this instrument were administered by specialized trauma psychologists. Furthermore, the IES-r has been demonstrated to have high concurrent validity and diagnostic accuracy (e.g., Creamer et al., 2003).

A further limitation is that we had no pre-exposure data with regard to any physical and mental complaints that were present before the attack. This is true for most disaster research as it is very difficult to systematically collect data on health measures and relevant predictors prospectively in a controlled way, benefitting from pre-event data. As a result, and our study is unfortunately no exception, disaster research should be careful with implying causality between exposure to a particular hazards or stressor on the one hand and observed health issues on the other as pre-existing vulnerabilities and health problems have been confirmed crucial predictors (Galea et al., 2008; Van der Velden and Wittmann, 2008; Yzermans et al., 2009; Bonanno et al., 2010; Shaikh et al., 2021). We were therefore dependent on self-report by the respondents to assess these. It is possible that pre-existing physical and mental problems may have affected outcomes. This is, however, very common in disaster health research. Pre-exposure survey data is almost never available. Moreover, it is complex and time-consuming to identify relatively small groups disseminated over multiple communities in health registry data.

Another limitation is that we cannot rule out that the intervention by the interviewers (advice offered to some of the respondents to help them find the right care and support) had a positive or negative influence on the health and well-being of the interviewees. On the other hand, it was a conditio *sine qua non* to conduct the monitor, as other approaches were not possible. The goal of aftercare programs is to offer care to those who need it. While some respondents may have not been able to access this care without help by the interviewers, in the end the outcome – leading those who need it to the right care – is the desired one. Another factor that may have influenced outcomes at the second round of interviews is the fact that roughly 1 year after the shooting – about 6 months before the interviews – the COVID-19 pandemic – started. This may have caused additional stressors for survivors, possibly impacting health outcomes. More anxiety, depression, stress or loneliness as a result of the pandemic were reported by a number of the affected.

A final limitation is that there was a high degree of dropout between the first and second round of interviews, with 8 out of 21 respondents declining participation in the second round. Those who dropped out initially graded their life on average much lower than full participants (3 vs. 6), and had a high score on the PTSD-measure. This indicated that the survivors with the most problems did not take part in the second round of interviews. Despite this, results from the second round of interviews show that the tram shooting still had a major impact on the health and wellbeing of those affected. We can only speculate that the overall impact at 18 months post-attack would have been even greater if this selective dropout had not occurred. Related to this, two additional respondents were added to the study through snowball sampling. As they were not known to victims support, they were not offered the same degree of aftercare as the others. Furthermore, they were deemed not to be victims in the legal sense, which led to frustration and grief. This may have impacted their recovery, and underscores how important it is to improve registration of victims.

### Implications for health service delivery

In addition to earlier mentioned implications regarding registration and monitoring of victims, the results of this study have implications for health service delivery after terrorist attacks and other disasters. It is clear that monitoring of the needs and problems of survivors is necessary beyond the first few months, as problems may develop or worsen at a later time. In addition, victims may not be immediately ready to accept care or realize that they might need it. When monitoring and aftercare related to the disaster is over, not everyone will be able to find their way to appropriate care through regular channels. This means that specialized aftercare should remain available for survivors at a later point in time. The problem is that typically, follow-up capacity is scarce, and that regular care channels might not always be equipped to deal with the specific problems of survivors of terrorist attacks or other disasters. A possible solution might be to provide survivors with a one-stop shop for disaster response services: a centralized (online) location for information and advice regarding psychosocial, practical and legal matters (e.g., Van Herpen et al., 2022). This platform can also be used for referring survivors to (specialized) care and can offer a forum where fellow survivors can meet. One could consider adding a professional trauma professional to this center; not to medicalize problems but to provide guidance, like the interviewers did in this study. When such a platform is embedded within existing care structures it can also help organizations to align their services (Van Herpen et al., 2022). While such a one-stop shop has been implemented incidentally in the Netherlands (Jacobs et al., 2019; Van Herpen et al., 2022) it is not yet standard practice, and was not implemented after the Utrecht tram shooting. The results of this study suggest that it could be wise to offer this platform after all major events and disasters. A smart way to achieve this might be to develop a standardized format which can be easily adapted to the current event.

## Conclusion

For a large proportion of survivors, the tram-shooting continued to have a long-term impact on mental and physical health and functioning: half of the survivors/affected still needed help in finding the right care 18 months post-attack. Results also demonstrate that health monitoring, aimed at offering the right care at the right time was impeded by challenges regarding registration of victims. These challenges were not new, and will occur again if registration of survivors and the exchange of their contact information are not improved. Finally, results show that health monitoring is only useful if appropriate care can be offered at the right time. Valuable steps in achieving this are: (1) provide survivors with a one-stop-shop where they can find information, be referred to care and meet fellow survivors, (2) tackle waiting lists in specialized care to make sure that the needed care is more readily accessible for victims and all others in need of such care.

## Data availability statement

Data are available from study authors upon reasonable request.

## Ethics statement

All study participants gave written informed consent, and were notified they could stop participation at any time. The study design was submitted to the Medical Ethics Review Committee from the Utrecht University Medical Centre (registration number W19.104), which decided that the study needed no further ethical approval.

## Author contributions

CP, CY, FS, and MD planned and designed the study. CP and FS coordinated the data collection and processed the data. MB wrote both first and consecutive drafts of the manuscript. All authors participated in data interpretation and provided input into the development of the manuscript, read, and agreed to the published version of the manuscript.

## Funding

The work of LES was funded by the Research Council of Norway, grant number 288321.

## Conflict of interest

The authors declare that the research was conducted in the absence of any commercial or financial relationships that could be construed as a potential conflict of interest.

## Publisher’s note

All claims expressed in this article are solely those of the authors and do not necessarily represent those of their affiliated organizations, or those of the publisher, the editors and the reviewers. Any product that may be evaluated in this article, or claim that may be made by its manufacturer, is not guaranteed or endorsed by the publisher.

## Footnotes

^1^While the Utrecht tram shooting was a relatively small terrorist attack, with only a single perpetrator and four fatalities, it is still classified as a terrorist attack. The reason for this is that perpetrator had a terrorist motive for conducting his attack. This was made clear during the attack by what the attacker shouted, and by a note left at the scene. The perpetrator was later convicted for multiple homicides, multiple attempted homicides and criminal threats, all with a terrorist motive. Nevertheless, the perpetrator of the tram shooting also suffered from a personality disorder and intellectual disability, as became clear after a psychological evaluation conducted during the trial. This is something that is seen in more terrorist attacks worldwide, especially in attacks by lone wolfs (Ho et al., 2019). In other words, the line between patient with a mental illness and a terrorist can be a fine one.

^2^The 24 October Square is the location of the attack.
